# Fucoidan Extracted from *Fucus vesiculosus* Ameliorates Colitis-Associated Neuroinflammation and Anxiety-like Behavior in Adult C57BL/6 Mice

**DOI:** 10.3390/md24010042

**Published:** 2026-01-14

**Authors:** Xiaoyu Song, Na Li, Xiujie Li, Bo Yuan, Xuan Zhang, Sheng Li, Xiaojing Yang, Bing Qi, Shixuan Yin, Chunxue Li, Yangting Huang, Ben Zhang, Yanjie Guo, Jie Zhao, Xuefei Wu

**Affiliations:** 1 Department of Medical Physiology, School of Basic Medical Science, Dalian Medical University, Dalian 116044, China; songxy0627@163.com (X.S.); 18739604855@163.com (X.L.); yuanb01@dmu.edu.cn (B.Y.); qibing0990@163.com (B.Q.); yshixuan0008@163.com (S.Y.); lichunxue2000@163.com (C.L.); huangyangting0913@163.com (Y.H.); 15667293341@163.com (B.Z.); 2 National-Local Joint Engineering Research Center for Drug-Research and Development (R&D) of Neurodegenerative Diseases, Dalian Medical University, Dalian 116044, China; lina0322@dmu.edu.cn (N.L.); zhangxuan201812@163.com (X.Z.); lisheng_1996@163.com (S.L.); yxjwewy2022@163.com (X.Y.); 3 Department of Basic Medical Science, Huanghuai University, Zhumadian 463000, China; 4 Department of Medical Technology, Xinglin College, Liaoning University of Traditional Chinese Medicine, Shenyang 110136, China; 5 Department of Pathogen Biology and Microecology (Pathogen Biology Laboratory), School of Basic Medical Science, Dalian Medical University, Dalian 116044, China

**Keywords:** IBD, DSS, fucoidan, gut–brain axis, neuroinflammation, microglia

## Abstract

Fucoidan, a complex sulfated polysaccharide derived from marine brown seaweeds, exhibits broad biological activities, including anticoagulant, antitumor, antiviral, anti-inflammatory and lipid-lowering effects. Fucoidan confers neuroprotection in animal models of a broad spectrum of brain disorders such as Parkinson’s disease (PD) and depression. However, the effect of fucoidan on gut-derived neuroinflammation and associated behavioral changes has been scarcely investigated. In comparison to fucoidan from other brown seaweeds, that from *Fucus vesiculosus* exhibited a better neuroprotective effect in vivo and more potent radical scavenging activity in vitro. Fucoidan from *Laminaria japonica* ameliorates behavioral disorders related to acute ulcerative colitis (UC) in aged mice. It is of interest to assess the effects of fucoidan administration on intestinal and brain inflammation in the acute colitis mouse model. Fucoidan treatment ameliorated DSS-induced intestinal pathology, reduced the inflammatory mediator expression in the gut and brain, and activated intestinal macrophages and cortical microglia in the UC mice. It also protected the intestinal mucosal barrier and blood–brain barrier as well as prevented neuronal damage, while alleviating anxiety-like behavior in UC mice. These results suggest fucoidan supplementation may help prevent brain disorders, such as depression and PD, potentially involving gut–brain axis-related mechanisms, as fucoidan suppresses gut-derived neuroinflammation.

## 1. Introduction

Inflammation is an innate immune response coordinated through the interaction of multiple cell types and signaling molecules that generate local and systemic reactions. The primary cell types involved are white blood cells and endothelial cells. The purpose of inflammation is to eliminate and control the initial stimulus; for instance, this occurs through phagocytosis and inflammasome activation, which trigger apoptosis and ultimately lead to tissue repair and scar formation [1]. However, inflammatory responses, especially chronic inflammation, can cause tissue damage and promote disease pathology. Neuroinflammation refers to diffuse immune activity within the central nervous system (CNS). CNS inflammation differs from peripheral inflammation in several ways, one key feature being the involvement of microglia and astrocytes as the main cellular components. Historically, the CNS was considered immune-privileged. However, despite the protection of the blood–brain barrier (BBB), a specialized barrier surrounding the brain, it is neither immunologically inert nor fully isolated from the peripheral immune system [2]. Neuroinflammation is instead a common pathological characteristic of many acute and chronic brain diseases. Excessive or prolonged neuroinflammation can devastate neuronal and overall brain function, thereby contributing to the onset and progression of neurodegenerative diseases, such as Alzheimer’s disease (AD) and multiple sclerosis (MS) [3]. Chronic inflammation is also strongly associated with schizophrenia and other psychiatric disorders [4].

Inflammatory bowel disease (IBD) is a chronic, recurrent intestinal inflammatory disorder caused by multifactorial etiologies, including Crohn’s disease and ulcerative colitis (UC). The precise cause of IBD remains unclear, but it involves complex interactions among genetic, environmental, microbial, and immune factors [5]. Behavioral comorbidities, such as anxiety and depression, are prominent in patients with IBD. A clinical study comparing healthy controls and patients with IBD found that those with symptomatic IBD exhibited the highest depression and anxiety scores, along with elevated intestinal IL-6, interleukin-1β (IL-1β), and serum IL-6 expression levels [6]. Additionally, individuals with IBD face a markedly higher risk of developing Parkinson’s disease (PD) compared with non-IBD populations [7]. Studies have shown that signals from the inflamed gut can affect the brain; specifically, in experimental colitis models, α4β7 integrin-mediated interactions between leukocytes and brain vascular endothelial cells are enhanced, leading to neuroimmune activation and anxiety-like behavior [8]. These findings suggest that targeting peripheral and intestinal inflammation is crucial to controlling brain inflammation and preventing neurodegenerative or psychiatric disorders.

Interactions among neurons, glial cells, and the immune system are essential for maintaining brain functions, including cognition, learning, and social behavior. Although glial cells are the principal cells involved in neuroinflammation, the stimuli that activate them often originate in the periphery. Research has demonstrated that disturbances in intestinal microbiota and elevated serum proinflammatory factors contribute to astrocyte activation in the brain [9]. Bidirectional communication between the gastrointestinal tract and brain forms the gut–brain axis. This axis regulates both brain and gastrointestinal function through neural, metabolic, and endocrine mediators, which are closely associated with inflammation [10]. Systemic chronic inflammation resulting from dysregulated intestinal microbiota primarily reflects excessive intestinal inflammation caused by immune dysfunction at the intestinal mucosal barrier. Such inflammation originates at the intestinal mucosal barrier interface or worsens following its disruption. Intestinal inflammation can promote chronic diseases along its signaling pathways and ultimately compromise BBB integrity, contributing to neuroinflammation, brain aging, and neurodegeneration [11]. Consequently, inhibiting glial cell overactivation and combating neuroinflammation have emerged as critical therapeutic strategies for CNS disorders, highlighting the central role of the gut–brain axis.

Fucoidan, also called sulfated fucans, is a complex sulfated polysaccharide derived from marine brown seaweed. In recent years, it has become a major focus of polysaccharide research [12]. It has been indicated that seaweed extracts and polysaccharides including fucoidan are effective candidates for the development of drugs, biological food additives, and functional nutrition products for the treatment and prevention of IBD [13]. The ameliorative effect of fucoidan administration on experimental colitis in mice has been attributed to the anti-inflammatory/antioxidant properties and the ability to modulate gut microbiota and metabolism of the polysaccharide [14,15]. In vitro and in vivo experimental evidence collectively verifies that fucoidan confers neuroprotection across diverse brain disorder models (e.g., AD, PD, MD, depression and cerebral ischemia) via multiple mechanisms: cholinergic system enhancement, functional preservation of BBB and mitochondria, inflammatory inhibition, and attenuation of oxidative stress and apoptosis [16]. However, the impact of fucoidan on gut-derived neuroinflammation and its associated behavioral changes remains largely unexplored. Moreover, the source, species, molecular weight, composition, and structure of fucoidan, as well as its route of administration, are crucial to its effects [17]. Xing et al. isolated and purified fucoidan from four sources, namely *Holothuria polii*, *Laminaria japonica*, *Ascophyllum nodosum* and *Fucus vesiculosus* (*F. vesiculosus*). Among these, the fucoidan derived from *F. vesiculosus* was found to exert the most prominent neuroprotective effect in the 1-methyl-4-phenyl-1,2,3,6-tetrahydropyridine (MPTP)-induced Parkinson’s disease (PD) mouse model [18]. Additionally, an in vitro study comparing the biological activities of fucoidan extracted from five brown seaweed species demonstrated that *F. vesiculosus*-derived fucoidan exhibited the strongest free radical scavenging activity [19]. We hypothesized that oral fucoidan from *F. vesiculosus* attenuates experimental colitis and associated neuroinflammation in adult mice.

Dextran sodium sulfate (DSS)-induced colitis is a well-established model for IBD, producing marked pathological changes in mouse and rat colons. Several plant-derived polysaccharides have shown efficacy in animal models of colitis or IBD. Using a preventive intervention design, we examined intestinal pathology, inflammatory mediators, BBB-related proteins, neuroinflammatory markers, neuronal integrity, and anxiety-like behavior. We found that oral fucoidan administration mitigated DSS-induced colitis and associated neuroinflammation/neuronal damage, suggesting that fucoidan may inhibit the transmission of peripheral inflammation to the CNS.

## 2. Results

### 2.1. Fucoidan Improved DSS-Induced Intestinal Pathology Changes in UC Model Mice

**Figure 1:**
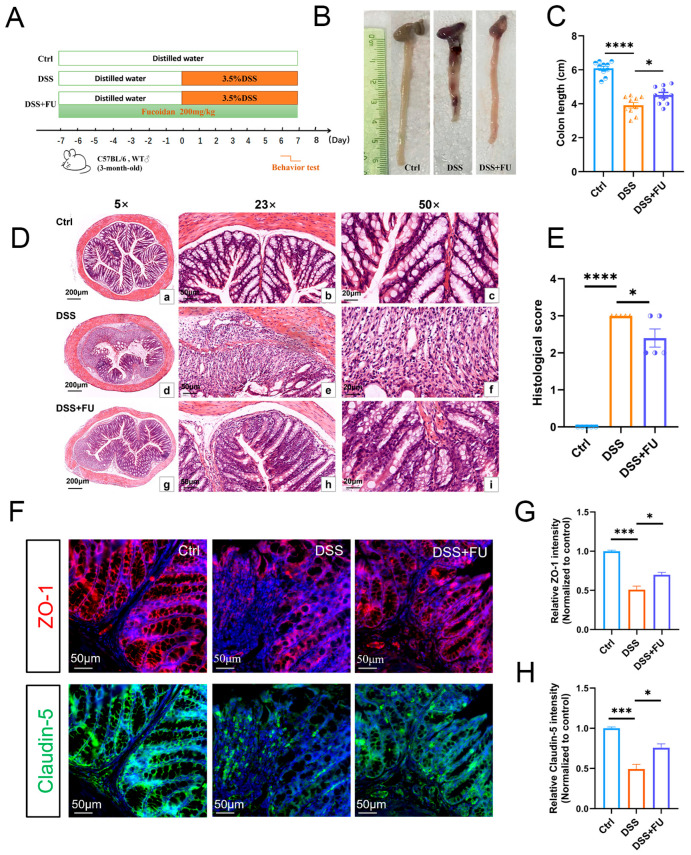
Fucoidan alleviated intestinal pathological changes caused by DSS. (**A**) Diagram of the experiment groups. (**B**,**C**) Measurement of Colon Length. Compared to the control group, the colon length in the DSS group was significantly reduced, which was alleviated by fucoidan treatment. (**D**,**E**) HE staining of colonic tissues from the control (**a**–**c**), DSS-induced (**d**–**f**), and fucoidan-treated (**g**–**i**) groups, shown at 5×, 23×, and 50× magnification, respectively. Histological scores assessing mucosal injury were significantly increased due to DSS treatment, while this increase was mitigated by fucoidan-treated. (**F**–**H**) Immunofluorescence staining of ZO-1 and Claudin-5 proteins in the colon. The decreased expression of ZO-1 and Claudin-5 induced by DSS was ameliorated by fucoidan treatment. Data are presented as mean ± SEM. (*) *p* < 0.05; (***) *p* < 0.001; (****) *p* < 0.0001.

Three groups were tested (Figure 1A): control, UC model (DSS), and fucoidan-treated (DSS+FU) groups. Colon tissues were collected after DSS treatment, and the full colon length colon (from the lower part of the ileocecal valve to the upper part of the rectum) was measured. Compared with the control, the DSS group had a significantly shorter colon length (6.1 ± 0.4 cm vs. 3.9 ± 0.5 cm). However, fucoidan treatment alleviated this shortening, with the DSS+FU group exhibiting a colon length of 4.5 ± 0.5 cm (Figure 1B,C). Compared with the control, colon length in the DSS group was significantly reduced, although this shortening was significantly improved in the DSS+FU group. H&E staining, performed to observe morphological changes in the intestinal mucosa (Figure 1D,E), showed that controls had intact mucosa with regularly arranged glands, whereas DSS-treated mice exhibited substantial inflammatory cell infiltration, severe mucosal injury, and disordered glands, with a significantly elevated intestinal mucosal injury score. Fucoidan-treated mice (DSS+FU group) showed partial repair, with significantly fewer inflammatory cells, more ordered glands, and a lower intestinal mucosal injury score compared with their DSS group counterparts. Immunofluorescence was conducted to examine the expression of intestinal mucosa barrier-related proteins, namely ZO-1 and claudin-5 (Figure 1F–H). Fucoidan treatment significantly reduced barrier damage compared with that observed in the DSS-treated group.

### 2.2. Fucoidan Mitigated Peripheral and Cerebral Inflammation in UC Mice: Secretion of Inflammatory Cytokines and Expression of Inflammatory Factor Genes

**Figure 2:**
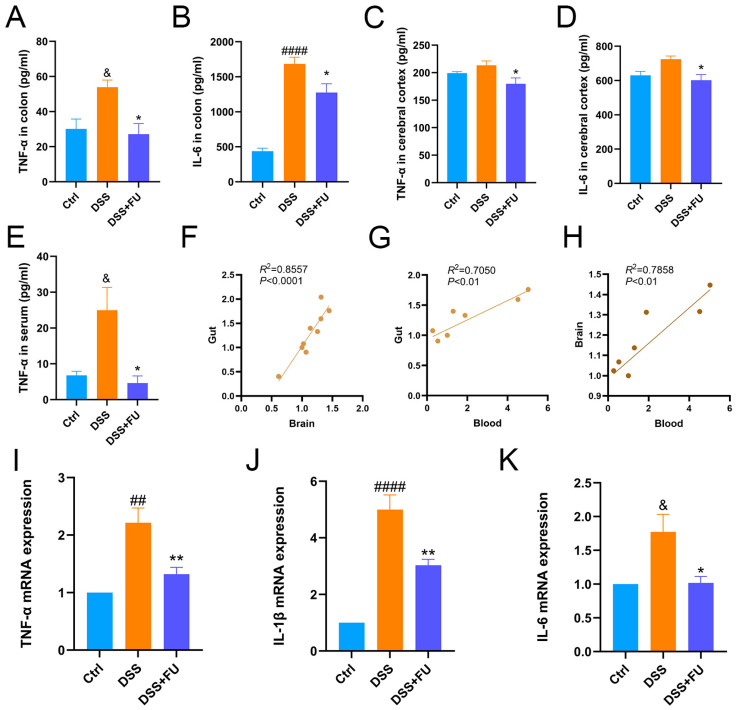
Fucoidan mitigated peripheral and cerebral inflammation induced by DSS: secretion of inflammatory cytokines. (**A**) Colon TNF-α levels. (**B**) Colon IL-6 levels. (**C**) Cerebral cortex TNF-α levels. (**D**) Cerebral cortex IL-6 levels. (**E**) Serum TNF-α levels. (**F**–**H**) Correlation analysis of TNF-α expression. Relative mRNA levels of TNF-α (**I**), IL-1β (**J**), and IL-6 (**K**) in the cortical cortex of mice. The increased gene expression induced by DSS was significantly decreased by fucoidan treatment. Data are presented as mean ± SEM, *n* = 5. (&) *p* < 0.05 compared with the Ctrl group; (##) *p* < 0.01 compared with the Ctrl group; (####) *p* < 0.0001 compared with the Ctrl group; (*) *p* < 0.05 compared with the DSS group. (**) *p* < 0.01 compared with the DSS group.

Using ELISA, proteins were detected in the serum, colon tissue, and cerebral cortex of mice. DSS increased TNF-α and IL-6 protein expression in colonic and cortical tissues, but these effects were significantly attenuated by fucoidan (Figure 2A–D). Serum TNF-α expression was also elevated by DSS and attenuated by fucoidan (Figure 2E). Correlation analyses of TNF-α levels were conducted between the serum and intestine, serum and brain, and brain and intestine (Figure 2F–H), indicating a statistical association between peripheral and cerebral inflammatory markers.

TNF-α, IL-6, and IL-1β mRNA levels in the cerebral cortex were measured via qPCR. DSS treatment elevated TNF-α, IL-1β, and IL-6 gene expression in the cortex (Figure 2I–K), but fucoidan significantly attenuated these increases. Thus, DSS induces neuroinflammation, whereas fucoidan markedly reduces this inflammation in UC model mouse brains.

### 2.3. Fucoidan Decreased NLRP3 Protein Expression in Colonic and Cortical Tissues of UC Model Mice

**Figure 3:**
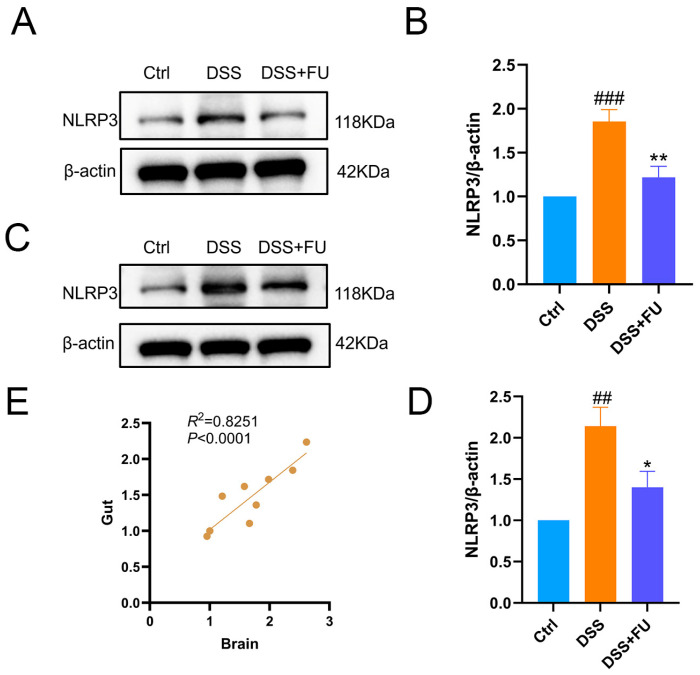
Fucoidan decreased NLRP3 protein expression in colonic and cerebral cortex tissues in UC mice. (**A**,**B**) The expression of NLRP3 protein in colon tissues. The increased expression of NLRP3 in the colon induced by DSS was significantly reduced by fucoidan. (**C**,**D**) The expression of NLRP3 protein in the cerebral cortex. The increased expression of NLRP3 in brain tissue induced by DSS was significantly reduced by fucoidan. (**E**) Correlation analysis of NLRP3 expression in the Gut and Brain. Data are presented as mean ± SEM, *n* = 4. (##) *p* < 0.01 compared with the Ctrl group; (###) *p* < 0.001 compared with the Ctrl group; (*) *p* < 0.05 compared with the DSS group; (**) *p* < 0.01 compared with the DSS group.

NOD-, LRR-, and pyrin domain-containing 3 (NLRP3) is a cytosolic innate immune receptor that assembles an inflammasome upon activation [20], leading to caspase-1–mediated proteolytic activation of IL-1β and downstream inflammatory responses [21]. Proteins extracted from colon and cortical tissue were analyzed for NLRP3 expression. DSS significantly increased NLRP3 expression in colonic (Figure 3A,B) and cortical (Figure 3C,D) tissues compared with the control, and fucoidan (DSS+FU group) significantly reduced this DSS-induced upregulation. Correlation analysis was conducted between colonic and cortical NLRP3 protein expression levels (Figure 3E). Here, inflammasome activation was not directly evaluated, given that neither caspase-1 activation nor IL-1β cleavage was measured. Nevertheless, the upregulation of NLRP3 was consistent with the elevated expression of proinflammatory cytokines in the brain and colon of DSS-treated mice, thereby supporting the involvement of an inflammatory process.

### 2.4. Fucoidan Inhibited Intestinal Macrophage and Brain Microglia Activation in UC Model Mice

**Figure 4:**
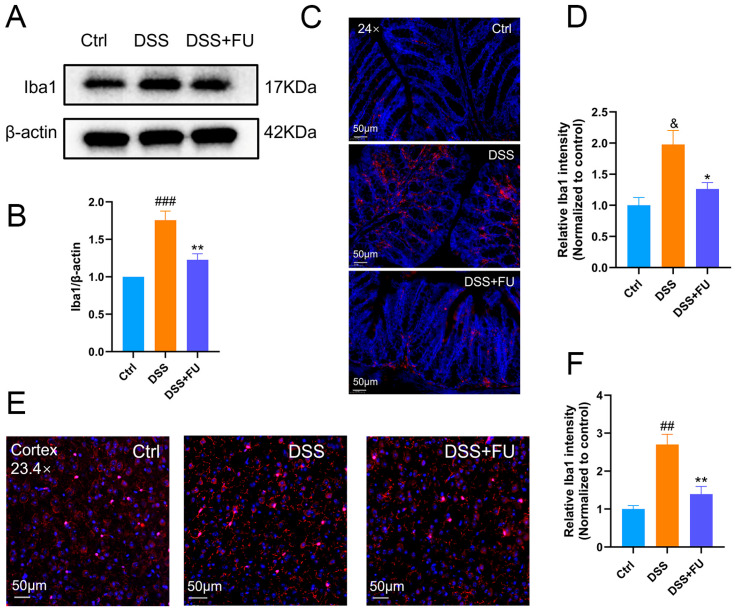
Fucoidan reduced macrophage activation in the intestinal tract and microglial activation in the brain of UC mice. (**A**,**B**) Expression of Iba-1 in the colon tissue of mice was detected using Western blot analysis. DSS-induced Iba-1 expression was suppressed by fucoidan treatment. (**C**,**D**) Immunofluorescence analysis of Iba1 positive cells in the colon of mice. (**E**,**F**) Immunofluorescence analysis of microglial cells in the cortex of mice. Immunofluorescence staining of Iba-1 in cortical sections of the brain demonstrated that DSS-induced microglial activation was attenuated by fucoidan. Data are presented as mean ± SEM. *n* = 4 for (**B**); *n* = 3 for (**D**,**F**). (&) *p* < 0.05 compared with the Ctrl group; (##) *p* < 0.01 compared with the Ctrl group; (###) *p* < 0.001 compared with the Ctrl group; (*) *p* < 0.05 compared with the DSS group. (**) *p* < 0.01 compared with the DSS group.

Microglial activation is an early and easily detectable hallmark of most neuropsychiatric, neuro-oncological, neurodevelopmental, neurodegenerative, and neuroinflammatory diseases [22]. Ionized calcium-binding adapter protein 1 (Iba1), encoded by the allograft inflammatory factor 1 gene, is an actin-interacting protein found in microglia. It has long been used as a cellular marker for microglia and macrophages, with increased Iba1 immunoreactivity, indicative of changes in microglial/macrophage status [23]. Western blotting was used to detect Iba1 expression in mouse colon tissue (Figure 4A,B), with immunofluorescence staining of Iba1-positive cells and microglia performed in colonic and cortical tissue (Figure 4C–F). Western blotting showed that DSS increased colonic Iba1 expression, which was significantly suppressed by fucoidan. Immunofluorescence staining of Iba1 in colon sections confirmed the Western blotting results. Furthermore, immunofluorescence staining of Iba1 in cortical sections showed that DSS-induced microglial activation was attenuated by fucoidan. Thus, fucoidan inhibits Iba1-positive cell activation in the intestine and microglial activation in the brain. Iba1 staining alone is insufficient to distinguish between the pro-inflammatory (M1) and anti-inflammatory (M2) microglial phenotypes. The elevated expression of proinflammatory cytokines in the brain and colon of DSS-treated mice supports the contribution of M1-type microglia, yet it does not preclude the involvement of M2-type cells.

### 2.5. Fucoidan Enhanced BBB Integrity in UC Model Mice

**Figure 5:**
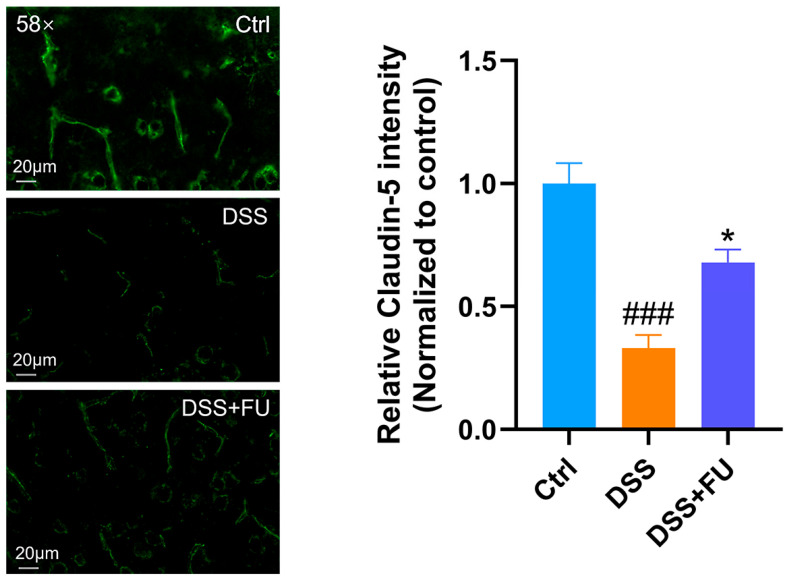
Fucoidan reduced the downregulation of BBB tight junction protein claudin-5 in UC mice. Immunofluorescence staining of claudin-5 in the cerebral cortex revealed that the decrease in claudin-5 levels caused by DSS was attenuated by fucoidan treatment. Data are presented as mean ± SEM. *n* = 3. (###) *p* < 0.001 compared with the Ctrl group; (*) *p* < 0.05 compared with the DSS group.

BBB integrity is crucial for CNS homeostasis. Structurally, the BBB is composed of endothelial cells that interact with pericytes, astrocytes, neurons, microglia, and perivascular macrophages within the neurovascular unit [24]. Claudin-5, a key protein in the tight junctions of vascular endothelial cells, plays a central role in regulating BBB integrity and permeability [25]. Increased expression of claudin-5 has been associated with neuroprotective effects in neurological diseases. Immunofluorescence staining for claudin-5 in mouse brain slices showed that a DSS-induced decrease in cortical claudin-5 expression was attenuated by fucoidan (Figure 5). Thus, fucoidan attenuated DSS-associated reductions in claudin-5 expression, a protein related to BBB structure. However, functional BBB permeability was not directly evaluated.

### 2.6. Fucoidan Alleviated Neuronal Damage in the Cerebral Cortex of UC Model Mice

**Figure 6:**
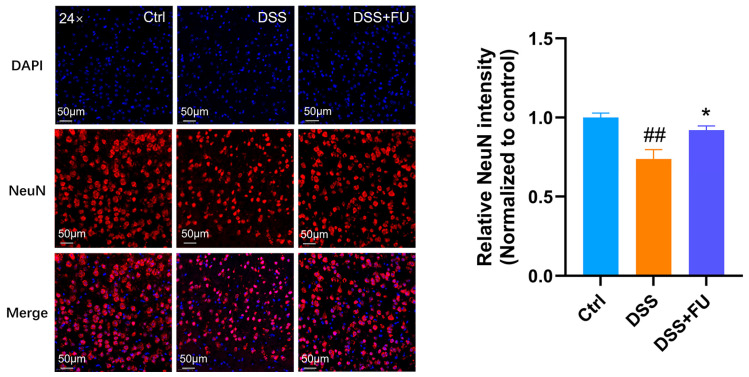
Fucoidan ameliorated the loss of NeuN expression in cerebral cortex of UC mice. Immunofluorescence staining of NeuN in the cortex. Compared with the control group, the number of NeuN^+^ neurons in DSS group was significantly reduced, which was prevented by fucoidan treatment. Data are presented as mean ± SEM. *n* = 3–4. (##) *p* < 0.01 compared with the Ctrl group; (*) *p* < 0.05 compared with the DSS group.

The human brain contains about 100 billion neurons [26]. NeuN is a well-established marker exclusively found in postmitotic neurons, initially identified through an immunological screen generating neuron-specific antibodies. Immunostaining has shown that NeuN is localized in the nuclei of mature neurons across most vertebrate brain regions [27]. NeuN and DAPI immunofluorescence were performed on brain sections from mice, with results showing that DSS-induced reductions in neuron numbers and cytoplasmic contraction were significantly alleviated by fucoidan (Figure 6). Therefore, fucoidan treatment was associated with preservation of NeuN immunoreactivity in the cerebral cortex, which suggests that fucoidan treatment might have neuroprotective effect.

### 2.7. Fucoidan Improved Anxiety-like Behavior in UC Model Mice

**Figure 7:**
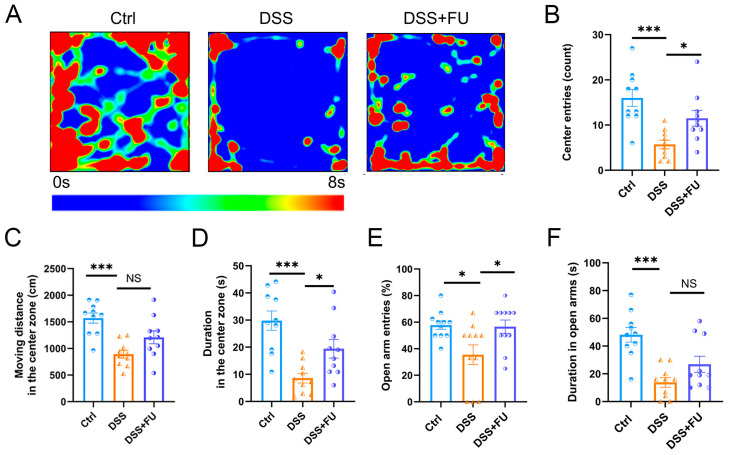
Fucoidan relieved anxiety-like behavior in UC mice. (**A**) Open field test. (**B**) Center entries. (**C**) Moving distance in the center zone. (**D**) Duration in the center zone. (**E**) Open arm entries. (**F**) Duration in open arms. Data are presented as mean ± SEM. *n* = 10. (*) *p* < 0.05; (***) *p* < 0.001; (NS) Not significant.

Behavioral tests were performed to further assess the impact of fucoidan on anxiety-related behavior (Figure 7). In the open field test (Figure 7A–D), DSS mice showed significantly fewer center entries and shorter time spent in the center zone compared with controls. In contrast, fucoidan (DSS+FU group) significantly reversed these changes (Figure 7B,D). In the elevated plus maze, DSS mice showed significantly fewer entries and a reduced proportion of time spent in the open arms compared with controls, whereas fucoidan (DSS+FU group) reversed these alterations (Figure 7E,F). These results indicate that fucoidan effectively improved DSS-induced anxiety-like behavior. Although these behaviors are commonly interpreted as anxiety-like, contributions from sickness-related behavior cannot be fully excluded.

## 3. Discussion

This study demonstrated the alleviative effect of fucoidan intervention on DSS-induced UC, colitis-associated neuroinflammation, and anxiety-like behaviors in adult mice. Beyond fucoidan’s impact on the peripheral system, including antitumor, anticoagulant, antithrombotic, antiviral, metabolic regulatory, and immunoregulatory effects [28], the therapeutic potential of fucoidan in brain disorders has also been investigated [29]. Notably, fucoidan from *L. japonica* protects dopamine neurons and improves motor dysfunction in several PD animal models [30,31,32,33,34], with mechanisms proposed to involve gut microbiota and gut–brain axis regulation [30]. Fucoidan can enter the bloodstream after oral administration and accumulate in organs, such as the kidneys [28]. However, one study reported that fucoidan from *F. vesiculosus* was not detectable in the mouse brain after tail vein injection [35]. As fucoidan is a macromolecular carbohydrate, it likely has difficulty crossing the BBB under normal conditions. Even when disrupted tight junctions permit limited penetration of fucoidan into the BBB, effective brain concentrations remain unlikely. Previous studies have shown that fucoidan attenuates acute UC in DSS-challenged mice by suppressing proinflammatory cytokines (TNF-α, IL-6, and IL-1β) and downregulating the NLRP3 inflammasome [36], thereby reducing intestinal inflammation and reinforcing mucosal barrier integrity [37]. Fucoidan has also been shown to regulate gut microbiota, correct abnormal intestinal flora structure, increase microbial diversity and abundance, and elevate the Bacteroides/Firmicutes ratio [38]. It is highly plausible that fucoidan modulated the gut microbiota composition, which in turn contributed to its protective effects against colitis and neuroinflammation in this study. The lack of gut microbiota profiling is a key limitation of the present study.

Neuroinflammation is a common feature of nearly all CNS diseases, and both systemic and intestinal inflammation contribute to neurodegenerative and psychiatric disease development [39]. Thus, compounds with anti-inflammatory or immunoregulatory properties can benefit the brain via the gut–brain axis. Our results indicate that fucoidan from *F. vesiculosus* can suppress both peripheral and neuroinflammation, which might be attributed to the gut microbiota modulation and the consequent changes in immune response and metabolism [15]. In addition, fucoidan is well recognized for its potent antioxidant properties [40], which may also account for its anti-inflammatory effects—given that oxidative stress and inflammation frequently coexist and mutually reinforce one another. Neuroinflammation may derive from both central (intracerebral) and peripheral sources. It represents a key contributor to neural injury and dysfunction, and its earliest and most common manifestations are anxiety or depressive symptoms, which can arise even when motor and cognitive functions remain intact. Anxiety-like behaviors are well-documented in DSS-induced colitis mice [37]. Fucoidan alleviated gut-derived inflammation and anxiety-like behaviors, suggesting that its neuroactivity may be partially mediated through gut–brain axis regulation. Consistently, a prior study showed that fucoidan from *L. japonica* ameliorates UC-related behavioral disorders in aged mice, restoring gut microbiota and reducing inflammation and neuronal damage. Together, these findings suggest that the anti-inflammatory effect of fucoidan is critical to its neuroactivity. However, fucoidan’s effects may differ depending on source, species, molecular weight, composition, and structure, especially regarding inflammation and immune modulation [17]. Further animal and human studies are needed to fully assess the anti-inflammatory potential of different fucoidan types. Another limitation of this study is the use of a preventive acute DSS-colitis model, without testing the long-term and therapeutic effect of fucoidan on colitis-related neuroinflammation. Further investigations into the impacts of species-specific fucoidans on chronic IBD and neuroinflammation models are highly warranted, as such studies would help clarify the clinical translational value of fucoidan.

A major challenge in developing drugs for CNS diseases is achieving sufficient BBB penetration [41]. One possible reason for drug development failure is focusing too heavily on drug structure–activity relationships while overlooking structure–drug exposure and selectivity relationships [42]. Importantly, the brain regulates peripheral organ function, whereas peripheral blood and tissues also influence CNS structure and function through peripheral nerves, immune cells, and humoral pathways. Neuroinflammation, a hallmark of nearly all CNS diseases, plays a key role in pathogenesis and may originate from peripheral inflammation, particularly in the gut [39]. Therefore, intestinal inflammation models can be valuable for screening drugs targeting peripheral inflammation without requiring BBB penetration, thereby helping prevent and treat CNS diseases. Microglia, as central mediators of neuroinflammation, respond to local cerebral signals and integrate inputs from the gastrointestinal tract [43]. Active signaling mechanisms between enteric glial cells and neurons also regulate gastrointestinal reflexes and can drive neuroinflammatory processes leading to long-term dysfunction [44]. Moreover, endothelial cells in the BBB not only participate in inflammatory processes but also regulate them [45]. During transitions from acute to chronic inflammation and innate to adaptive immunity, endothelial cells undergo marked phenotypic changes. Inflammatory mediators acting on endothelial cells frequently also target leukocytes, and many anti-inflammatory therapies influence endothelial cell behavior and function [46]. Fucoidan’s protective effect on BBB tight junction proteins may result from anti-inflammatory activity, although other regulatory mechanisms could contribute. Although other studies have proved the leaky BBB in colitis model [47], we failed to evaluate the BBB permeability in this study. Future studies will evaluate BBB function, immune cell adhesion to vascular endothelial cells, their entry into brain tissue, and their relationship with neuronal injury and function.

In conclusion, this study shows that fucoidan administration was associated with attenuation of DSS-induced intestinal and neuroinflammatory changes and behavioral alterations in adult mice. Its potential mechanisms involve multitarget modulation of the gut–brain axis. Specifically, fucoidan improves DSS-induced inflammatory changes in the intestine, peripheral blood, and brain, including reducing intestinal pathology and inflammation, lowering inflammatory factor levels in the blood, suppressing microglial activation and neuroinflammation in the cerebral cortex, preserving BBB tight junction protein expression, and mitigating neuronal damage and anxiety-like behavior. Reduction in intestinal inflammation and its spread to the brain through systemic humoral pathways may underlie fucoidan’s anti-neuroinflammatory effects. Overall, fucoidan represents a promising therapeutic candidate for managing UC and its associated neuropsychiatric comorbidities. Further mechanistic studies are required to establish causal pathways.

## 4. Materials and Methods

### 4.1. Reagents

Fucoidan, extracted from *F. vesiculosus* (product number: F8190; lot number: 0000175520; ≥95% purity) was purchased from Sigma-Aldrich Trading Co., Ltd. (Shanghai, China). Dextran Sulfate Sodium Salt, Colitis Grade was purchased from MP Biomedicals Co., Ltd. (Shanghai, China). DSS had a molecular weight of 36,000–50,000 Da and a 99% purity level.

### 4.2. Animal Treatment and Behavioral Testing

Three-month-old male C57BL/6J mice (SPF Animal Laboratory, Dalian Medical University, *n* = 30) were used in the study. All animals were acclimatized for 7 days under standard conditions (22 ± 2 °C and a 12/12-h light/dark cycle) with free access to water and standard chow. Experimental procedures followed the National Institutes of Health Guide for the Care and Use of Laboratory Animals and were approved by the Institutional Ethics Committee of Dalian Medical University (approval no. XL250704128). Acute UC was induced with 3.5% DSS solution administered ad libitum for 5 days. Fucoidan (200 mg/kg) was administered via oral gavage once daily for 14 days, consisting of a 7-day pretreatment period before DSS administration and a 7-day treatment period during DSS exposure (the fucoidan dose and pretreatment schedule were selected based on previous DSS-colitis studies demonstrating anti-inflammatory efficacy). Animals were randomly assigned to three groups (*n* = 10 per group): control (administered saline via gavage and provided with pure drinking water), UC model (administered saline via gavage and provided with a 3.5% DSS drinking solution), and fucoidan-treated (administered fucoidan via gavage and provided with a 3.5% DSS drinking solution).

After 5 days of 3.5% DSS administration, mice underwent the open field test and elevated plus maze test on days 6 and 7. Anxiety is thought to suppress animals’ drive to explore a novel environment, while for vice versa, less anxious animals are predicted to explore comparatively more. In the open field, exploration has been previously quantified by measures such as total locomotion or number of vertical rearing. Further, thigmotaxis in the open field, namely the tendency to stay in proximity of the walls while avoiding the centre of the arena, is often recorded and interpreted as a proxy for anxiety [48]. In the open field test, each animal was placed in the same arena corner and observed for 5 min. Behavioral videos were processed using rapid video editing software. Locomotor activity and movement trajectories were tracked and analyzed using EthoVision XT 11.5 (Noldus Information Technology, Wageningen, The Netherlands). Anxiety was assessed according to center entries, duration in the central zone, and distance moved in the center. Similar to the open-field test, the elevated plus maze test is based on the conflict between the exploration of a new environment and the natural aversion of rodents to bright and open spaces [48]. The elevated plus maze experiment was performed under double-blind conditions. Each animal was placed in the maze center and observed for 3 min. The number of entries in the open and closed arms, as well as the total time spent in the open arms, were recorded. Data were analyzed using GraphPad Prism 8.0.

### 4.3. Primary and Secondary Antibodies

The following antibodies were used: anti-β-actin (ab6276, Abcam, Shanghai, China), anti-Iba1 (ab178846, Abcam), anti-NeuN (ab177487, Abcam), anti-ZO-1 (ab221547, Abcam), anti-Claudin-5 (ab131259, Abcam), anti-NLRP3 (SC06-23, HUABIO, Hangzhou, China), Goat anti-Rabbit IgG (H+L) Cross-Adsorbed Secondary Antibody, Alexa Fluor™ 488 (A32731, Thermo Fisher Scientific, Waltham, MA, USA), and Goat anti-Mouse IgG (H+L) Highly Cross-Adsorbed Secondary Antibody, Alexa Fluor™ Plus 555 (A32727, Thermo Fisher Scientific).

### 4.4. Western Blot

Proteins from the colon and cerebral cortex were extracted using lysis solution buffer [tissue weight (mg):1 × RIPA (μL):PMSF (μL):protease inhibitors (μL):phosphatase inhibitors A and B (μL) = 10:100:1:1:1], and protein content was measured using the BCA protein assay (Keygen Biotech, Nanjing, China). A 5× SDS buffer was added to each 100-μL sample at a 4:1 ratio, and samples were incubated at 100 °C for 5 min to denature proteins. Proteins (30 μg per sample) were separated on 4–12% or 4–20% gradient gels.

Proteins were transferred to polyvinylidene difluoride (0.45 μm/0.22 μm) membranes, blocked with 5% BSA in 1× TBST for 1 h, and incubated overnight at 4 °C with primary antibodies. The next day, the membranes were washed three times in TBST, incubated with horseradish peroxidase-labeled secondary antibody for 1 h, and washed again three times in TBST. Infrared band signals were detected using SCG-W3000 PLUS gel analysis software (version 3.3.2, Servicebio, Wuhan, China). Densitometric analysis of immunoreactivity was conducted using Image J (1.8.0) software.

### 4.5. Quantitative Real-Time Polymerase Chain Reaction

Total RNA was extracted from cortex tissue using Trizol Reagent (Thermo Fisher Scientific, Carlsbad, CA, USA). RNA was then reverse-transcribed into cDNA using HiScript II Q RT SuperMix (Vazyme, Nanjing, China) for quantitative real-time polymerase chain reaction; according to the manufacturer’s instructions. A Bio-Rad CFX Manager with ChamQ Universal SYBR qPCR Master Mix (Vazyme, Nanjing, China) was used to perform qPCR. Gene expression was normalized to β-actin. Relative expression was calculated using the 2-ΔΔCt method. Primers and temperature cycles for RT-qPCR are shown in Supplementary Tables S1 and S2.

### 4.6. Histopathological Analysis

Colon tissues were fixed with 4% paraformaldehyde solution (Bioss, Beijing, China) for 2 days, dehydrated, cleared, paraffin-embedded, sectioned at 5-μm thickness, and stained with hematoxylin and eosin (H&E). Scan images were obtained using the 3D Histech Digital Pathology System (3DHistech Ltd., Budapest, Hungary). Pathological scores were based on inflammatory cell infiltration: 0, normal intestinal villi with intact intestinal mucosa and no infiltration; 1, inflammatory cells in the epithelial layer and lamina propria with mild hyperemia, edema not beyond the submembrane layer, and mild gland damage; 2, focal inflammation in the submucosa with substantial hyperemia and edema as well as moderate gland damage; 3, submucosal inflammatory infiltration with epithelial necrosis, large and deep ulcers possibly involving the muscle layer, and severe mucosal gland damage.

### 4.7. Immunofluorescence

Colon and brain tissues were fixed in paraformaldehyde for 24 h, dehydrated in 15% and 30% sucrose gradients, embedded in OCT, and rapidly frozen in liquid nitrogen until the embedding blocks solidified. Tissue sections (10 μm) were prepared using a Leica CM1850 Cryostat (Leica Biosystems, Nussloch, Germany) and mounted on antiadhesive slides. Sections were dried, washed three times with 0.3% PBST, and subjected to antigen retrieval for 25 min. They were then blocked with 3% BSA for 1 h, incubated overnight with primary antibodies, incubated with secondary antibodies for 1 h, and mounted using one drop of antifade mounting medium (Coolaber, Beijing, China) before a coverslip was applied. Scan images were obtained using the 3D Histech Digital Pathology System, and colocalization was analyzed using ImageJ (1.8.0) software.

### 4.8. ELISA

TNF-α and IL-6 levels in colon and cerebral cortex tissues, as well as TNF-α levels in serum, were measured using ELISA kits (Elabscience, Wuhan, China) following manufacturer instructions. The plate was read immediately at 450 nm on a microplate reader (Thermo Fisher Scientific, Waltham, MA, USA), and the concentration was determined by calculating formula from a standard curve. The experiment was performed three times. Data were analyzed via GraphPad Prism, with group differences evaluated using one-way ANOVA.

## Data Availability

The data presented in this study are available on request from the corresponding author.

## Supplementary Materials

The following supporting information can be downloaded at: https://www.mdpi.com/article/10.3390/md24010042/s1, Table S1. Primer sequences; Table S2. Temperature program used for RT-qPCR. Figure S1. Western blots for NLRP3 (colon tissue, *n* = 4); Figure S2. Western blots for NLRP3 (cerebral cortex, *n* = 4); Figure S3. Western blots for Iba1 (colon tissue, *n* = 4).

## Abbreviations

The following abbreviations are used in this manuscript:

AD	Alzheimer’s disease
IBD	Inflammatory bowel disease
UC	Ulcerative colitis
PD	Parkinson’s disease
DSS	Inflammatory bowel disease
CNS	Central Nervous System
BBB	Blood brain barrier
TNF-ɑ	Tumor necrosis factor alpha
IL-1	Interleukin-1β
IL-6	Interleukin-6
Iba1	Ionized calcium binding adapter molecule 1
NLRP3	NOD-, LRR-, and pyrin domain-containing 3
NeuN	Neuronal Nuclei
ZO-1	Zonula Occludens-1
