# Supplementary material for: Fucoidan Extracted from Fucus vesiculosus Ameliorates Colitis-Associated Neuroinflammation and Anxiety-like Behavior in Adult C57BL/6 Mice
Source: Mar Drugs. 2026 Jan 14;24(1):42. doi: 10.3390/md24010042 (PMC12843082; doi:10.3390/md24010042)

Table S1. Primer sequences

Gene	Forward primer (5′–3′)	Reverse primer (5′–3′)
β-actin	CACGATGGAGGGGCCGGACTCATC	TAAAGACCTCTATGCCAACACAGT
IL-1β	GCAGTGGTTCGAGGCCTAAT	CTCATCACTGTCAAAAGGTGGC
TNF-α	CGTCAGCCGATTTGCTATCT	CGGACTCCGCAAAGTCTAAG
IL-6	TCCATCCAGTTGCCTTCTTGG	CCACGATTTCCCAGAGAACATG

Table S2. Temperature program used for RT-qPCR.

Operation	Temperature (℃)	Time	Cycles
Initial activation	95	3 min	1
Denaturation	95	15 sec	35
Annealing	60	15 sec	
Extension	72	60 sec	
Final Extension	72	5 min	1

Figure S1. Original Western blots for NLRP3 (colon tissue, n=4 biological replicates).

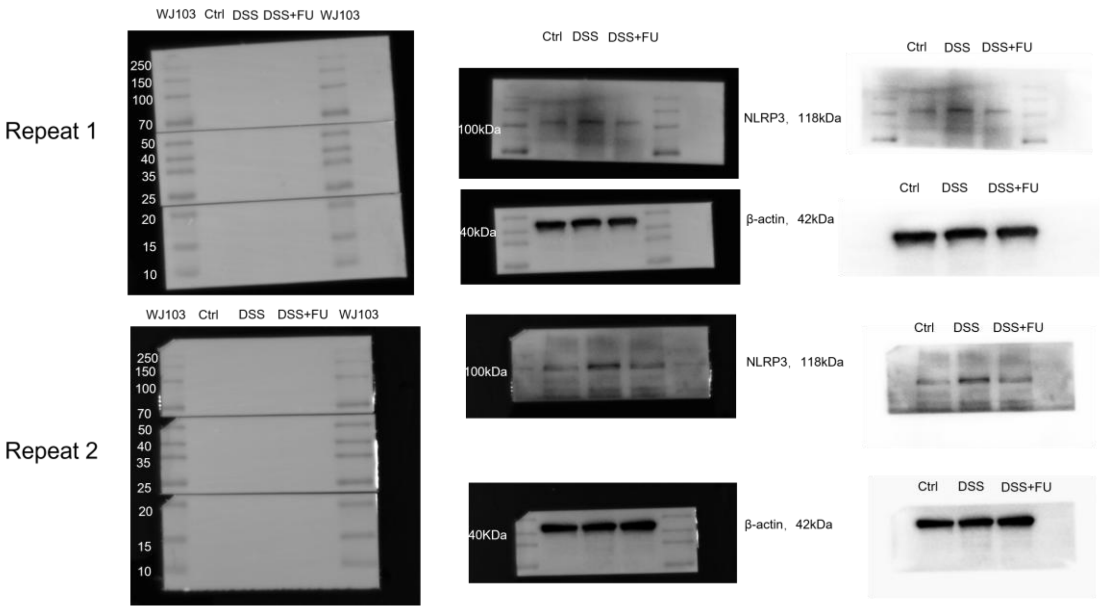

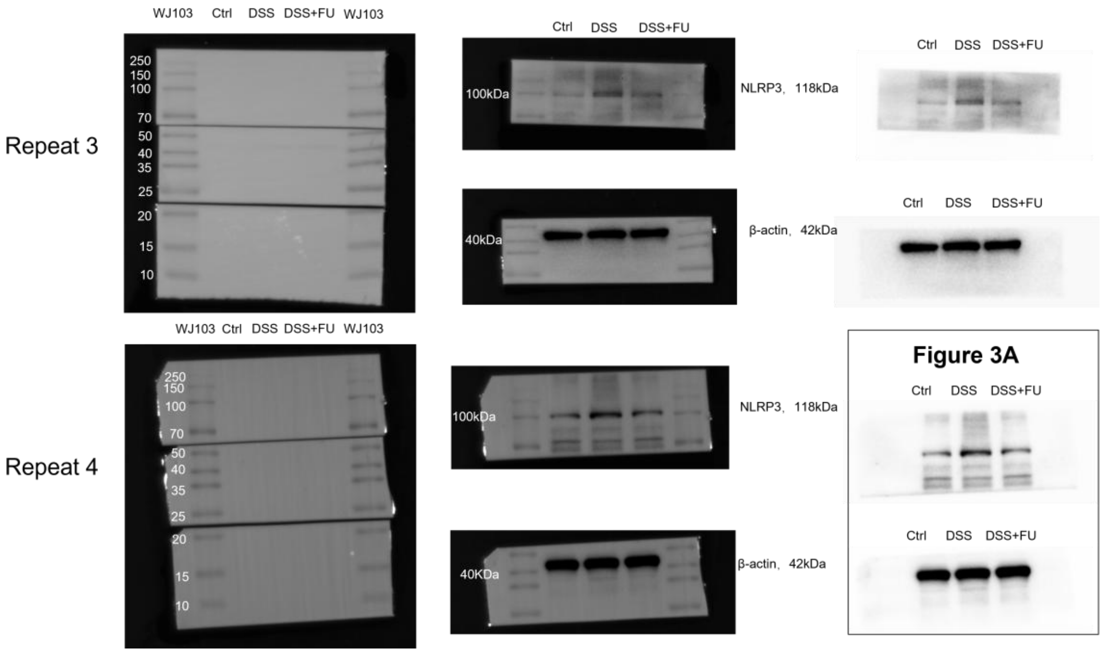

Figure S2. Original Western blots for NLRP3 (cerebral cortex, n=4 biological replicates).

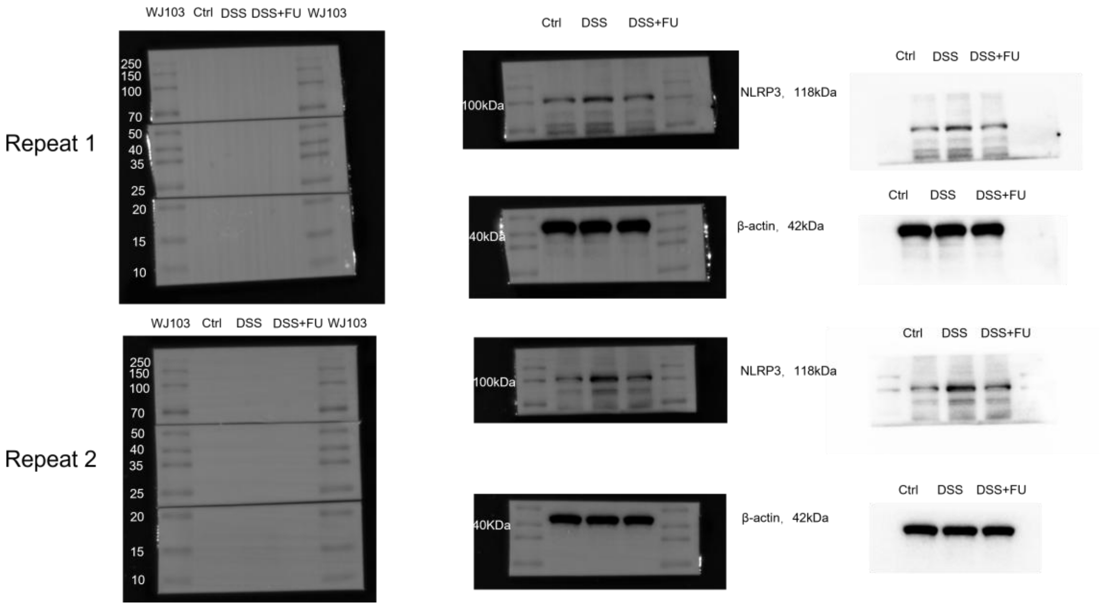

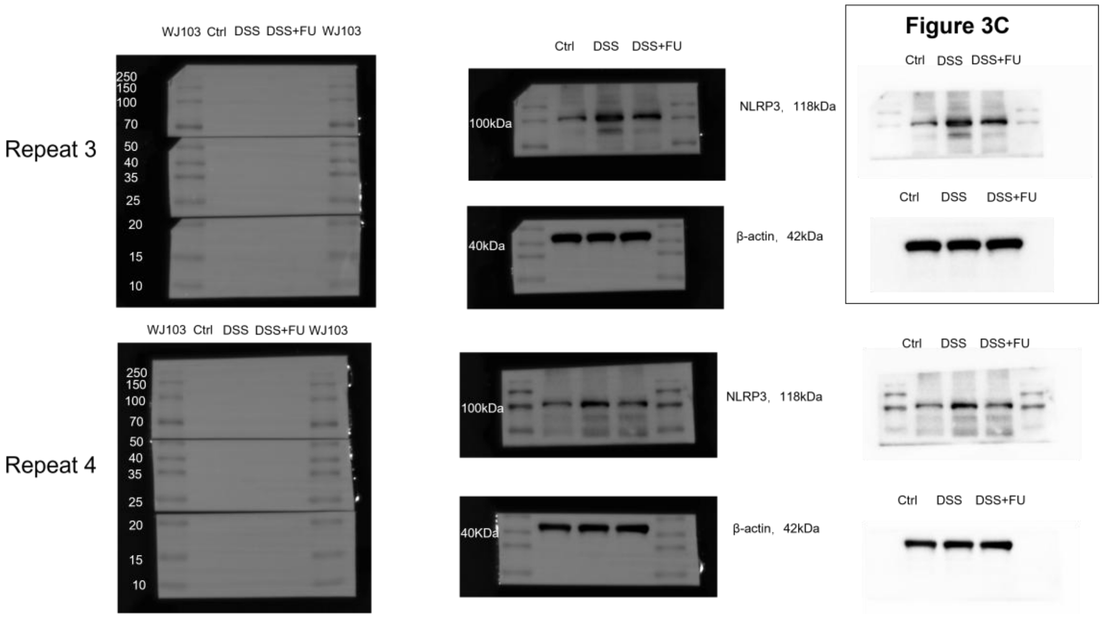

Figure S3. Original Western blots for Iba1 (colon tissue, n=4 biological replicates).

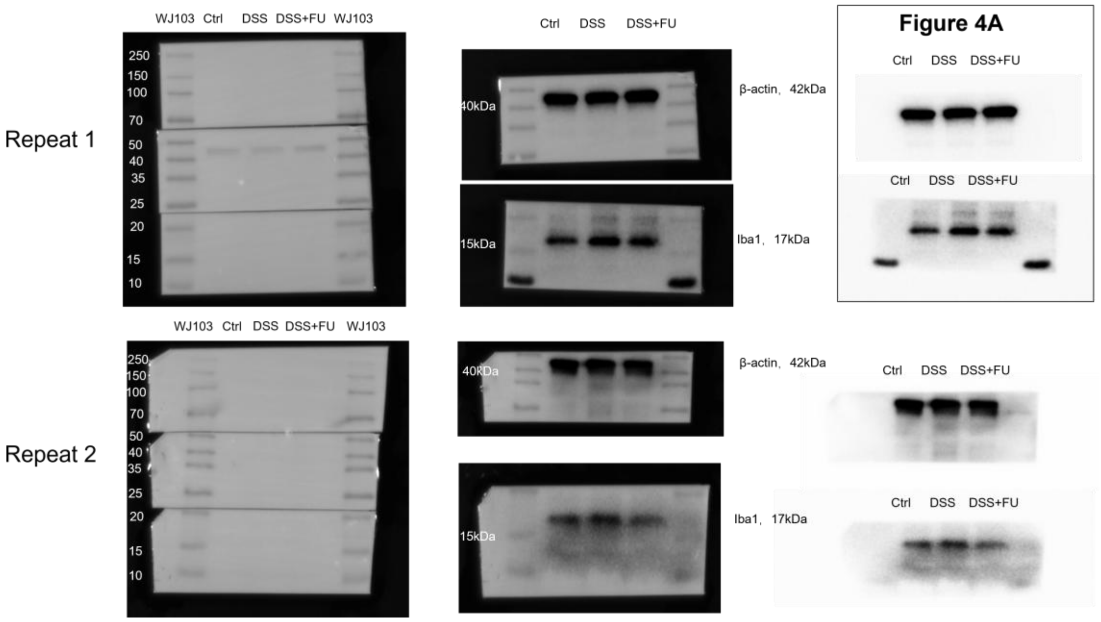

Repeat 3

Repeat 4

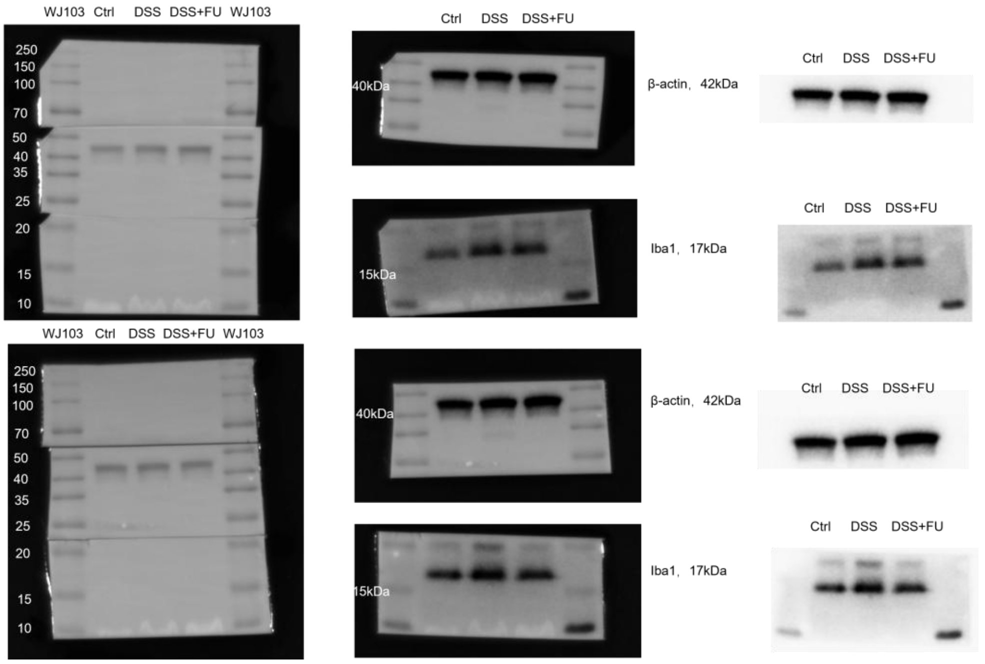

Supplement: Supplementary file 1 [file marinedrugs-24-00042-s001.zip › marinedrugs-4065213-supplementary.pdf]
